# Small‐Molecule Sarco/Endoplasmic Reticulum Ca^2+^‐ATPase Activators Reverse Methylglyoxal‐Induced Inhibition through Nonantioxidant Mechanisms

**DOI:** 10.1002/cmdc.202500968

**Published:** 2025-11-30

**Authors:** Carlos Cruz‐Cortés, Silvia Micháliková, Petronela Rezbáriková, L. Michel Espinoza‐Fonseca, Jana Viskupičová

**Affiliations:** ^1^ Center for Arrhythmia Research Department of Internal Medicine Division of Cardiovascular Medicine University of Michigan Ann Arbor MI 48109 USA; ^2^ Centre of Experimental Medicine Institute of Experimental Pharmacology & Toxicology Slovak Academy of Sciences 84104 Bratislava Slovakia

**Keywords:** ABTS, activators, calcium, diabetes, DPPH, endoplasmic reticulum, lipid peroxidation, methylglyoxal, sarco/endoplasmic reticulum Ca^2+^‐ATPase

## Abstract

Impaired endoplasmic reticulum (ER) Ca^2+^ homeostasis contributes to *β*‐cell dysfunction under diabetic stressors such as methylglyoxal (MGX), a reactive byproduct that induces oxidative protein modifications and advanced glycation end‐products. The calcium pump sarco/endoplasmic reticulum Ca^2+^‐ATPase (SERCA), essential for ER Ca^2+^ regulation, is inhibited by MGX‐mediated carbonylation and thiol oxidation. Pharmacological SERCA activation has emerged as a promising strategy to restore ER Ca^2+^ balance, but whether protection results from direct allosteric modulation, indirect antioxidant effects, or both has remained unclear. Herein, it is shown that novel, potent synthetic activators directly stimulate SERCA and restore its activity following MGX‐induced inhibition. While some compounds display antioxidant activity, recovery of SERCA function correlated with activation potency rather than radical scavenging or lipid peroxidation inhibition. It is demonstrated for the first time that direct SERCA activation alone is sufficient to significantly reverse oxidative damage, revealing a mechanistically distinct therapeutic approach to preserve ER Ca^2+^ homeostasis in diabetes.

## Graphical abstract

Endoplasmic reticulum (ER) Ca2+ dysregulation drives β‐cell dysfunction under diabetic stressors like methylglyoxal, which inhibits the sarco/endoplasmic reticulum Ca2+‐ATPase (SERCA) pump through oxidative modifications. We show that synthetic SERCA activators restore pump activity after methylglyoxal‐induced damage. Their protective effects reflect direct activation potency rather than antioxidant effects, revealing SERCA stimulation as a distinct therapeutic strategy for preserving ER Ca2+ balance in diabetes.

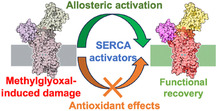

## 1 Introduction

Metabolic diseases, such as type 2 diabetes mellitus (T2DM) and obesity, are rising at an alarming rate, posing significant public health and economic challenges worldwide.^[^ ^1^ ^]^ The global prevalence of diabetes has doubled since 1990, with over 800 million adults currently affected worldwide.^[^ ^2^ ^]^ Diabetes accounted for approximately €170 billion in health expenditures in Europe in 2021, and in 2022, it was estimated that the cost of diagnosed diabetes in the US was about $307 billion in direct medical costs alone^[^ ^3^ ^]^ with an overall cost of $966 billion globally.^[^ ^4^ ^]^ T2DM is driven by insulin resistance, *β*‐cell dysfunction, and chronic hyperglycemia, while obesity acts as a major risk factor by promoting inflammation, dyslipidemia, and systemic metabolic stress. Together, these conditions increase the risk of cardiovascular, renal, and neurological complications, contributing substantially to morbidity and mortality. Despite advances in glucose‐lowering drugs and lifestyle interventions, current therapies remain insufficient, as they largely address symptoms rather than the underlying molecular drivers of disease. Thus, identifying mechanisms and therapeutic strategies that target the cellular basis of metabolic dysfunction is essential to curb the growing global impact of T2DM and obesity.^[^ ^1^ ^]^

The T2DM progression is driven by elevated levels of circulating glucose and its metabolites, free fatty acids, and pro‐inflammatory cytokines, leading to glucolipotoxicity, ER‐stress induction, *β*‐cell apoptosis, and functional decline.^[^ ^5^ ^]^ Among these stressors, methylglyoxal (MGX) plays a key role in the pathogenesis of diabetes and its complications. MGX is a highly reactive dicarbonyl compound formed under hyperglycemic conditions, and it is a major contributor to glycation of proteins, lipids, and DNA, and the subsequent formation of advanced glycation end‐products (AGEs).^[^ ^6^ ^]^ In proteins, MGX‐mediated modifications occur primarily through nucleophilic addition to Arg, Lys, and Cys residues, with Arg and Lys adducts leading to irreversible AGE formation.^[^ ^7^ ^]^ MGX and MGX‐derived AGEs are implicated not only in diabetes and its vascular complications but also in other age‐related chronic inflammatory diseases, including cardiovascular disease, cancer, and neurodegeneration.^[^ ^8^ ^]^ Therefore, reversing the detrimental effects of MGX represents a promising therapeutic avenue to mitigate MGX‐associated conditions. Indeed, current experimental therapeutic strategies to counteract MGX toxicity include glyoxalase inducers, glycation inhibitors, MGX scavengers, and antioxidants that counteract oxidative stress and AGE formation.^[^ ^9^ ^]^ Hypoglycemic agents, such as metformin, teneligliptin, and pioglitazone, provide *β*‐cell protection by enhancing antioxidant defenses, reducing glucotoxicity, suppressing CD36 expression, and stabilizing mitochondrial function.^[^ ^10^ ^]^

The sarco/endoplasmic reticulum Ca^2+^‐ATPase (SERCA) is an essential ion pump responsible for maintaining intracellular Ca^2+^ homeostasis. By actively transporting Ca^2+^ from the cytosol into the sarcoplasmic/endoplasmic reticulum (SR/ER), SERCA contributes to the clearance of more than 70% of cytosolic Ca^2+^ in most mammalian cell types.^[^ ^11^ ^]^ This activity is vital for the regulation of Ca^2+^‐dependent signaling, contractility, and cell survival. Under oxidative stress, SERCA undergoes irreversible oxidative post‐translational modifications, notably cysteine oxidation, tyrosine nitration, and carbonylation, that impair pump activity and promote cellular dysfunction and disease progression. Critical modifications linked to SERCA inactivation involve sulfonylation at Cys674^[^ ^12^ ^,^ ^13^ ^]^ and nitration at Tyr294/295,^[^ ^14^, ^15^, ^16^ ^]^ both of which have significant functional implications for muscle physiology, cardiovascular and metabolic conditions, as reviewed by Viskupicova and Espinoza–Fonseca.^[^ ^17^ ^]^ Accumulating evidence indicates that MGX compromises SERCA activity through enzyme carbonylation and oxidation of free thiol groups, leading to inhibition of the pump's ATPase activity and impaired Ca^2+^ transport efficiency.^[^ ^18^ ^]^ Carbonylation primarily involves the covalent modifications of lysine and arginine residues, forming stable carbonyl adducts. In SERCA2a, carbonylation of four critical residues (Arg164, Lys476, Lys481, and Arg636) correlated with reduced ATPase activity and altered Ca^2+^ handling in diabetic models.^[^ ^19^ ^,^ ^20^ ^]^ This evidence underscores the importance of oxidative and carbonyl modifications in SERCA dysfunction and highlights the need for strategies that restore the pump's activity and maintain Ca^2+^ homeostasis under pathological conditions.

In recent years, pharmacological activation of SERCA has emerged as a promising therapeutic strategy to restore Ca^2+^ homeostasis and mitigate ER stress under pathological conditions. For example, allosteric SERCA activators have shown efficacy in preclinical models of diabetes and metabolic conditions.^[^ ^21^, ^22^, ^23^ ^]^ In cardiac contexts, SERCA activators have shown particular promise for improving contractility and reducing pathological remodeling in cardiomyopathy and heart failure.^[^ ^24^, ^25^, ^26^ ^]^ Despite these advances, a critical gap in the field is the absence of direct mechanistic evidence that pharmacological SERCA activation can restore enzymatic activity once the protein has been functionally compromised by MGX. Therefore, it remains unclear whether the beneficial effects of SERCA activators stem from direct allosteric modulation of the enzyme, indirect antioxidant activity, or a combination of both mechanisms. Addressing this question is essential for defining the therapeutic scope of SERCA activators and for rationally guiding their development in disorders characterized by MGX‐induced SERCA inhibition. To address this question, we evaluated a series of novel synthetic small‐molecule SERCA activators^[^ ^27^ ^]^ for their ability to stimulate the ATPase activity of SERCA after MGX damage. We examined whether recovery of enzymatic activity arises predominantly from direct allosteric activation of the pump or from the antioxidant properties of the compounds. By dissecting these mechanisms, we demonstrate for the first time that small‐molecule SERCA activators restore pump function after MGX damage through direct activation, independent of antioxidant effects.

## 2 Results and Discussion

We first evaluated fifteen synthetic molecules (**Table** **1**)^[^ ^27^ ^]^ for their ability to activate SERCA1a. To maintain consistency with our previously published study, the compound numbering in this work begins at compound 33. Some numbers are omitted to preserve continuity with earlier compound designations. These compounds were selected because they activate cardiac SERCA2a with EC_50_ values below 10 µM, exhibit sigmoidal concentration–response relationship with no loss of activity or inhibition observed at compound concentrations up to 50 µM, and span a wide range of cLogD_7.4_ values,^[^ ^27^ ^]^ allowing us to rule out assay interference as a confounding factor and determine whether SERCA1a ATPase activation correlates with lipophilicity. All compounds were tested at a concentration of 10 µM for ATPase activity using SERCA1a, and activity is represented as the % of the change in maximal velocity (*V*_max_) relative to the activity of untreated SERCA. A summary of the SERCA1a ATPase activities and cLogD_7.4_ values is shown in Table 1. We found that all fifteen molecules tested here activate SERCA1a, increasing the pump's activity by 13–33%. Among the compounds tested, we found that the tetrahydroquinoline **33**, the indoles **42** and **48**, and the benzofurans **51** and **54** showed the highest efficacy, activating SERCA1a ATPase activity by ≈30%. These findings are consistent with our previous study demonstrating that indoline and benzofuran derivatives are potent activators and further reveal that their high efficacy is maintained across both SERCA1a and SERCA2a isoforms. Notably, we found no correlation between stimulation of SERCA1a activity and cLogD_7.4_ (*r* = 0.08, Figure S1 of the Supplementary Information), indicating that compound efficacy is independent of lipophilicity at physiological pH, and unlikely to result from assay interference. These results highlight the broad isoform efficacy of this chemotype and establish a foundation for testing its ability to reverse MGX‐induced SERCA1a inhibition.

**Table 1:** Effects of the compounds (10 µM) on the ATPase activity of SERCA1a and their calculated cLogD_7.4_ values. Data are expressed as % of vehicle control (mean ± SEM, *n* = 3–5).

Compound	Chemical structure	Normalized SERCA1a activity (%)	cLogD_7.4_
**33**	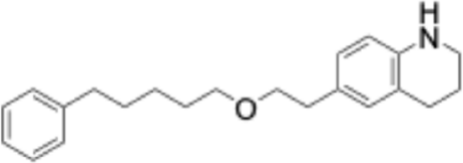	130 ± 2	5.36
**34**	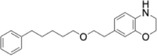	115 ± 3	4.55
**36**	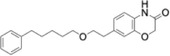	117 ± 6	4.27
**42**	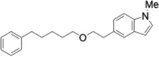	132 ± 4	5.81
**44**	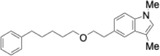	121 ± 4	6.32
**47**	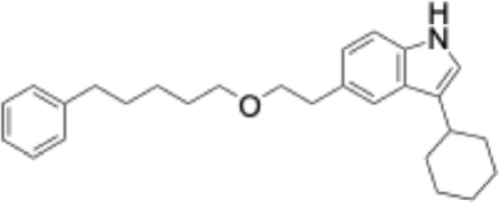	116 ± 3	7.70
**48**	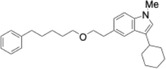	128 ± 5	7.92
**49**	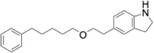	115 ± 3	4.95
**50**	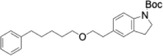	113 ± 1	6.30
**51**	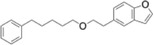	133 ± 6	5.64
**52**	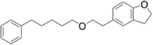	117 ± 3	5.13
**53**	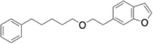	114 ± 0	5.64
**54**	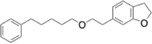	130 ± 3	5.37
**55**	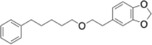	124 ± 5	5.11
**56**	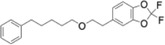	115 ± 4	6.50

**Figure 1:**
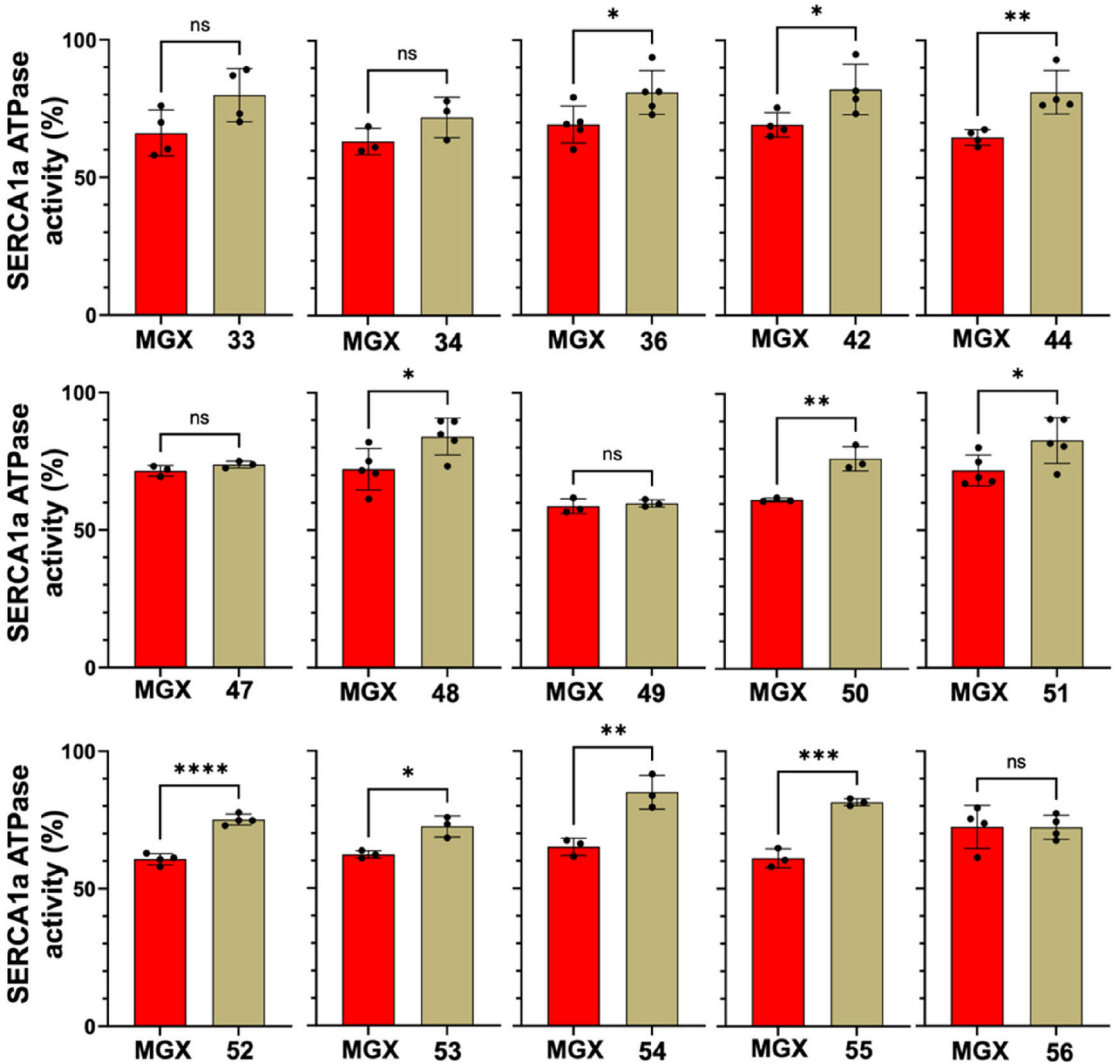
Protection of SERCA1a ATPase activity from MGX‐induced inhibition by small‐molecule activators. SERCA1a ATPase activity was measured after exposure to methylglyoxal (MGX; 4 mM) in the absence (red bars) or presence of fifteen small‐molecule activators tested at a concentration of 10 µM (tan bars). Values are expressed as percent ATPase activity relative to untreated SERCA1a controls (mean ± SEM, *n* = 3–5). Statistical comparisons were performed using unpaired two‐tailed t‐test; *p < 0.05, **p < 0.01, ***p < 0.001, ****p < 0.0001, ns = not significant.

**Figure 2:**
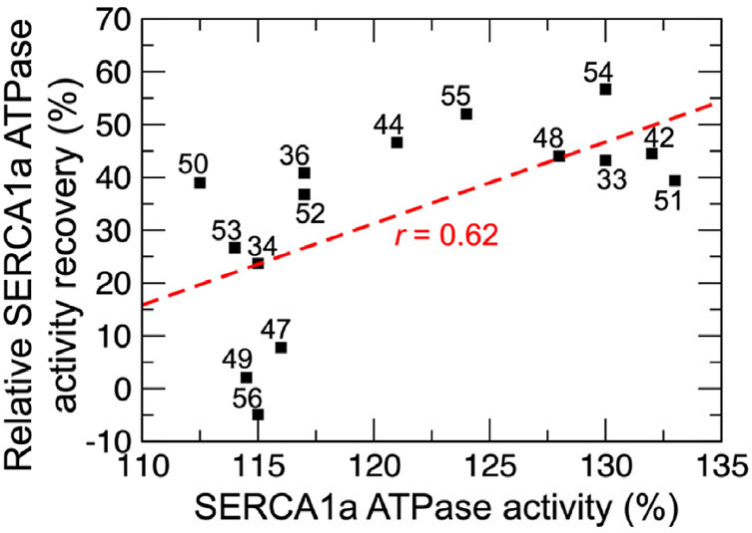
Correlation between SERCA1a activation and protection against MGX‐induced inhibition. Scatter plot of SERCA1a ATPase activation (expressed as % activity relative to untreated control) versus recovery of SERCA1a ATPase activity following MGX treatment (expressed as % relative recovery). Each data point represents an individual compound tested at a concentration of 10  µM. A linear regression model (red‐dashed line) was applied to evaluate the correlation between activation of ATPase activity and relative recovery (*r* = 0.62).

We next assessed the effects of the fifteen validated activators on the ATPase activity of SERCA1a following MGX treatment. To this end, SERCA1a was incubated with MGX in the presence or absence of each activator, and enzymatic activity was quantified. In agreement with previous studies,^[^ ^18^ ^,^ ^28^ ^]^ we found that MGX treatment markedly reduced SERCA1a ATPase activity, lowering the *V*_max_ of the pump by 30–45%. Relative to MGX‐treated SERCA1a, twelve of the compounds were able to restore pump activity at a concentration of 10 µM (**Figure** **1**). Several activators were able to prevent the inhibitory effects of MGX; in particular, the dihydroisoquinolinone **36**, the indoles **42, 44,** and **48**, the indoline **50**, the benzofurans **51, 52,** and **54**, and the benzodioxole **55** restored SERCA1a activity by up to ≈57% relative to MGX‐treated controls (Figure 1). We note that compounds **47** and **48**, although highly hydrophobic and not considered viable drug candidates, were included as mechanistic probes to assess whether hydrophobicity influences the recovery of SERCA1a activation following MGX treatment. Nevertheless, we found no direct correlation between recovery of MGX‐treated SERCA1a activity and cLogD_7.4_ (*r* = –0.17, Figure S2 of the Supplementary Information). These findings demonstrate that the stimulatory effects of these compounds on SERCA1a's ATPase activity are preserved even under conditions of oxidative stress. This observation is particularly notable when compared with our previous work on polyphenolic SERCA1a activators, including resveratrol, gingerol, and ellagic acid.^[^ ^28^ ^]^ While these natural products were able to protect SERCA1a from MGX‐induced inhibition, most required high concentrations (>50 µM) to achieve measurable protection.^[^ ^28^ ^]^ Interestingly, correlation analysis showed that SERCA1a activation was positively and linearly associated with recovery of MGX‐induced damage (*r* = 0.62, *p* = 0.0132; **Figure** **2**). These findings suggest that compounds with higher activation potency generally confer greater recovery of SERCA1a activity after MGX‐induced inhibition. Overall, our results demonstrate that most of the validated activators not only stimulate SERCA1a activity but also effectively counteract MGX‐induced inhibition. Several chemotypes, including indoles, benzofurans, and a benzodioxole, restored pump function, highlighting their ability to maintain efficacy under oxidative stress conditions at much lower concentrations compared to natural compounds.^[^ ^28^ ^]^

Our results indicate that direct activation of SERCA1a is likely the primary mechanism driving recovery following MGX‐induced inhibition. However, additional protective effects, such as enzyme stabilization under oxidative stress or interactions with the membrane, may also contribute to the observed recovery. Notably, prior studies have suggested that the protective effects against MGX‐induced SERCA1a damage may arise from the strong antioxidant properties of the compounds rather than from direct stimulation of SERCA1a catalytic activity.^[^ ^28^ ^]^ To clarify the mechanism, we evaluated the activators in three independent antioxidant assays (DPPH•, ABTS•^+^, and C11‐BODIPY^581/591^) to determine whether their restorative effects are primarily due to direct SERCA1a activation, an antioxidant activity, or a combination of both. The DPPH is a commonly used antioxidant assay that primarily measures hydrogen atom transfer from an antioxidant to the DPPH• radical, while the ABTS•^+^ assay quantifies both electron and hydrogen atom transfer and offers a broader radical‐scavenging scope with greater solvent versatility.^[^ ^29^ ^]^ Additionally, we used the biologically relevant C11‐BODIPY^581/591^ lipid peroxidation assay to detect chain‐breaking antioxidant activity through a ratiometric red‐to‐green fluorescence shift (581/591 nm → 488/520 nm), to directly measure lipid peroxidation in SR membranes. Most activators tested here exhibited little to no radical‐scavenging activity in DPPH• and ABTS•^+^ assays at 10 µM. We found that, at a concentration of 10  µM, only compounds **33**, **34**, **47,** and **49** produced strong ABTS•^+^ scavenging activity, while compounds **33**, **34,** and **49** had a mild DPPH• scavenging activity (**Table** **2**). Notably, compounds **33**, **34**, **47,** and **49** displayed antioxidant activity comparable to the synthetic standard Trolox in the ABTS•^+^ assay (Table 2). Similarly, compounds **33**, **34**, and **49** strongly inhibited lipid peroxidation in SR vesicles at a compound concentration of 10 µM (Table 2). All tested activators share a benzyloxy‐alkyl–heteroaromatic scaffold; however, compounds **33**, **34**, and **49** contain a secondary amine linked to an electron‐rich heteroaromatic core, enabling hydrogen donation and stabilization of radicals through resonance and electron delocalization, which accounts for their strong antioxidant properties.

**Table 2:** ABTS•^+^ and DPPH• radical scavenging activity and inhibition of lipid peroxidation by tested compounds (10 µM) compared with the reference antioxidant trolox. Radical scavenging (%) was determined from the decrease in absorbance of ABTS•^+^ at 734 nm (5 min, RT) and DPPH• at 517 nm (30 min, RT). Lipid peroxidation inhibition in BODIPY 581/591‐labeled sarcoplasmic reticulum membranes was assessed after 30 min incubation with AAPH (10 mM) at 37 °C. Data are expressed as mean ± SD (*n* = 3).

Compound	ABTS scavenging (%)	DPPH scavenging (%)	Inhibition of lipid peroxidation (%)
**33**	77.0 ± 1.4	9.5 ± 3.1	33.9 ± 8.1
**34**	43.9 ± 1.3	5.6 ± 4.1	50.4 ± 6.0
**36**	−1.3 ± 0.3	−5.1 ± 3.6	−6.3 ± 9.7
**42**	0.4 ± 0.6	−4.8 ± 2.6	7.8 ± 4.2
**44**	11.5 ± 2.7	−2.8 ± 0.9	−5.6 ± 12.9
**47**	33.6 ± 2.1	−3.0 ± 0.7	1.6 ± 8.7
**48**	0.6 ± 0.5	−5.4 ± 2.5	−15.8 ± 9.7
**49**	60.9 ± 3.2	11.3 ± 2.6	38.3 ± 6.5
**50**	−1.0 ± 0.3	−5.3 ± 2.5	−13.5 ± 10.6
**51**	−1.4 ± 0.1	−5.6 ± 3.1	−16 ± 2.4
**52**	−1.1 ± 0.4	−3.2 ± 0.7	−7.9 ± 1.5
**53**	−2.1 ± 0.1	−5.0 ± 3.0	−9.9 ± 0.3
**54**	−0.5 ± 0.2	−5.1 ± 2.8	−2.2 ± 6.7
**55**	−0.5 ± 0.6	−4.8 ± 2.8	1.5 ± 11.2
**56**	−2.2 ± 0.2	−4.7 ± 2.9	−10.8 ± 3.1
**Trolox**	37.1 ± 3.2	16.7 ± 1.0	72.7 ± 4.3

**Figure 3:**
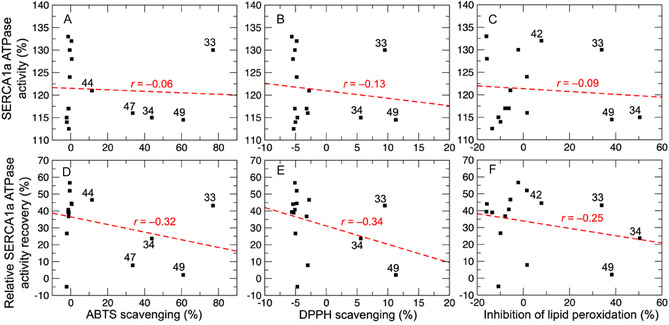
Correlation between SERCA1a activation or recovery from MGX‐induced inhibition and antioxidant activities. A–C) Scatter plots of SERCA1a ATPase activity (% relative to untreated control) versus ABTS scavenging, DPPH scavenging, and inhibition of lipid peroxidation. D–F) Scatter plots of recovery of MGX‐inhibited SERCA1a activity (% relative recovery) versus ABTS scavenging, DPPH scavenging, and inhibition of lipid peroxidation. Linear regression (red dashed line) was used to assess correlations between SERCA1a functional effects and antioxidant activity.

Finally, we investigated whether the antioxidant capacity of the activators contributes to either direct stimulation of SERCA1a or protection against MGX‐induced inhibition. To this end, we compared SERCA1a ATPase activity and activity recovery with ABTS•^+^ and DPPH• radical scavenging, as well as inhibition of lipid peroxidation. We did not observe positive correlations between radical scavenging or lipid peroxidation inhibition and either SERCA1a activation (*r* = −0.06 to −0.13; **Figure** **3**, panels A‐C) or recovery of activity following MGX treatment (*r* = −0.25 to −0.34; Figure 3, panels D–F). These weak negative correlations indicate that radical‐scavenging activity is neither sufficient nor predictive of the ability of activators to stimulate SERCA1a or restore its function under oxidative stress. Indeed, despite their high radical‐scavenging and inhibition of lipid peroxidation capacity, compounds **33**, **34**, and **49** provided only modest recovery of SERCA1a activity, underscoring that antioxidant effects alone do not account for protection against MGX‐induced inhibition. Conversely, several activators with low antioxidant activity were effective at stimulating and restoring ATPase function, supporting the notion that direct SERCA1a activation, rather than general antioxidant mechanisms, is the predominant driver of functional recovery.

In summary, our findings demonstrate that novel small‐molecule activators effectively stimulate SERCA1a and restore its activity following MGX‐induced inhibition. While certain compounds exhibit strong antioxidant properties due to structural features enabling hydrogen donation and radical stabilization, antioxidant capacity did not correlate with either direct SERCA1a activation or recovery after oxidative damage. Instead, recovery of the pump's ATPase activity strongly correlated with activation potency, indicating that direct allosteric stimulation of SERCA1a, rather than radical scavenging or lipid peroxidation inhibition, underlies functional rescue. These findings are significant because they reveal for the first time that direct SERCA activation is sufficient to reverse MGX‐induced inhibition of SERCA1a's ATPase activity. Our data support the idea that pharmacological SERCA activation could counteract ER stress by directly correcting Ca^2+^ mishandling in *β*‐cells, a central defect in T2DM pathophysiology. Furthermore, the ability of this chemotype to maintain efficacy across SERCA isoforms suggests broad therapeutic utility in muscle, cardiac, and metabolic tissues. Ultimately, pharmacological SERCA activation represents a promising and mechanistically distinct therapeutic strategy to preserve Ca^2+^ homeostasis and cellular function, offering a foundation for the development of first‐in‐class interventions for diabetes, obesity, and associated metabolic disorders.

## 3 Experimental Section

3.1

3.1.1

##### Chemicals

Unless stated otherwise, all chemicals were of an analytical grade and purchased from Sigma (St. Louis, MO, USA) and Thermo‐Fisher Scientific (Waltham, MA, USA) unless otherwise indicated. Methylglyoxal solution (≈40% in H_2_O), 2,2‐diphenyl‐1‐picrylhydrazyl, 2,2′‐azino‐bis(3‐ethylbenzothiazoline‐6‐sulfonic acid, 2,2′‐azobis(2‐methylpropionamidine) dihydrochloride, were obtained from Sigma (St. Louis, MO, USA). BODIPY 581/591 C11 (Lipid Peroxidation Sensor) was purchased from Thermo‐Fisher Scientific (Waltham, MA, USA). The fifteen small‐molecule SERCA activators used in this study belong to a library of molecules recently published by us.^[^ ^27^ ^]^ The compounds used are: 6‐(2‐((5‐Phenylpentyl)oxy)ethyl)−1,2,3,4‐tetrahydroquinoline hydrochloride (**33**); 7‐(2‐((5‐Phenylpentyl)oxy)ethyl)−3,4‐dihydro‐2H‐benzo[b][1,4]oxazine hydrochloride (**34**); 7‐(2‐((5‐Phenylpentyl)oxy)ethyl)‐2H‐benzo[b][1,4]oxazin‐3(4H)‐one (**36**); 1‐Methyl‐5‐(2‐((5‐phenylpentyl)oxy)ethyl)‐1H‐indole (**42**); 1,3‐Dimethyl‐5‐(2‐((5‐phenylpentyl)oxy)ethyl)‐1H‐indole (**44**); 3‐Cyclohexyl‐5‐(2‐((5‐phenylpentyl)oxy)ethyl)‐1H‐indole (**47**); 3‐Cyclohexyl‐1‐methyl‐5‐(2‐((5‐phenylpentyl)oxy)ethyl)‐1H‐indole (**48**); 5‐(2‐((5‐Phenylpentyl)oxy)ethyl)indoline hydrochloride (**49**); *tert*‐Butyl 5‐(2‐((5‐phenylpentyl)oxy)ethyl)indoline‐1‐carboxylate (**50**); 5‐(2‐((5‐Phenylpentyl)oxy)ethyl)benzofuran (**51**); 5‐(2‐((5‐Phenylpentyl)oxy)ethyl)−2,3‐dihydrobenzofuran (**52**); 6‐(2‐((5‐Phenylpentyl)oxy)ethyl)benzofuran (**53**); 6‐(2‐((5‐Phenylpentyl)oxy)ethyl)−2,3‐dihydrobenzofuran (**54**); 5‐(2‐((5‐Phenylpentyl)oxy)ethyl)benzo[*d*][1,3]dioxole (**55**); and 2,2‐Difluoro‐5‐(2‐((5‐phenylpentyl)oxy)ethyl)benzo[*d*][1,3]dioxole (**56**). The purity of the fifteen synthetic SERCA activators was > 95% (by HPLC).

##### Isolation of SERCA1a‐Enriched Microsomes

Isolation was performed as previously described.^[^ ^30^ ^]^ Briefly, rabbit fast‐twitch muscles were collected immediately after euthanasia and kept in ice until processing. Approximately 50 g of muscle was minced and blended in 150 mL of a buffer containing 100 mM KCl , 2.5 mM K_2_HPO_4_, 2.5 mM KH_2_PO_4_, and 2 mM EDTA (pH = 7.4), and a protease inhibitor cocktail (Sigma, St. Louis, MO). The mixture was centrifuged at 6,400 g for 20 min at 4 °C to remove a major part of the extracellular connective tissue. The obtained supernatant was filtered and centrifuged at 9,700 g for 20 min at 4 °C to remove the final remnants of cell debris. The filtrate was collected and centrifuged at 47,800 g for 60 min at 4 °C; the pellet was resuspended and homogenized using a Teflon homogenizer in 150 mL of a solution containing 1 M sucrose and 50 mM KCl. The suspension was centrifuged at 4,300 g for 30 min at 4 °C. The supernatant obtained was mixed and incubated for 1 h with a buffer containing 2 M KCl and 100 mM Mg^2+^‐ATP. The preparation was centrifuged at 84,500 g for 90 min at 4 °C, and the pellet was resuspended and homogenized in a 50 mM KCl solution. The suspension was centrifuged at 84,500 g for 90 min at 4 °C, and the pellet was resuspended in a solution that contained 5 mM HEPES, and 300 mM sucrose. The protein concentration of microsomes was determined with a Pierce Coomassie plus assay kit (Thermo‐Fisher Scientific, Waltham, MA). The estimated protein concentration in the microsomes was 2.4–3.8 mg ml^–^^1^. The microsomes were aliquoted, flash‐frozen in liquid nitrogen, and stored at –80 °C until use.

##### ATPase Activity Assay

We performed SERCA1a activity assays using an enzyme‐coupled NADH‐linked ATPase activity assay described previously.^[^ ^30^ ^]^ Briefly, we measured the activity of Ca^2+^‐ATPase in μmol min^−1^ mg^−1^ from the decrease in absorbance of NADH at 340 nm at 25 °C in a 96‐well format using a Synergy H1 (BioTek, Winooski, VT) microplate reader. Each well contained a 200 μL final volume of assay buffer containing SERCA1a buffer (50 mM MOPS, 100 mM KCl, 5 mM MgCl_2_, and 1 mM EGTA, pH = 7), 5 U lactate dehydrogenase, 5 U pyruvate dehydrogenase, 1 mM phosphoenolpyruvate, 5 mM ATP, 0.2 mM NADH, 0.5 μg of microsomal suspension, 2 μM of Ca^2+^ ionophore A23187, and seven free Ca^2+^ concentrations. The compounds (10 μM) with or without methylglyoxal (4 mM) were incubated for 30 min at 25 °C with the reaction mixture. The final free Ca^2+^ concentrations were calculated using MaxChelator and were achieved by using twelve individual stock CaCl_2_ solutions. Each plate included untreated microsomes (negative control), as well as microsomes treated with thapsigargin (inhibition control). To account for biological variability, we used microsomal fractions obtained from different isolations. In all cases, the maximal activity (*V*_max_) values were normalized relative to the untreated microsomes. Relative Ca^2+^‐ATPase activity recovery was calculated as the percentage of activity restored by a given compound relative to the activity lost after MGX treatment, as follows

(1) $$\%\;\text{recovery}=\left(\frac{\text{activity}\left(\text{compound}+\mathrm{MGX}\right)-\text{activity}\left(\mathrm{MGX}\right)}{\text{activity}\left(\text{control}\right)-\text{activity}\left(\mathrm{MGX}\right)}\right)\times 100$$

##### 2,2‐Diphenyl‐1‐Picrylhydrazyl (DPPH^•^) Assay

The DPPH^•^ (2,2‐diphenyl‐1‐picrylhydrazyl) radical scavenging activity of compounds was evaluated according to Prieto et al.^[^ ^31^ ^]^ with absorbance measured at 517 nm. Trolox was used as a reference antioxidant compound. Briefly, a 0.2 mM DPPH^•^ stock solution was prepared in 96% ethanol. Stock solutions of test compounds (10 mM in DMSO) were serially diluted in 96% ethanol to obtain final concentrations ranging from 0.8 to 100 µM. For the assay, 100 µL of DPPH^•^ solution was mixed with 100 µL of the test compound solution, and the decrease in absorbance was recorded after 30 min of incubation in the dark.

Antioxidant activity was expressed as % DPPH scavenging, calculated as

(2) $$\text{\% scavenging}=\left(1-\frac{A\left(\text{sample}\right)-A\;\left(\text{background}\right)}{A\left(\mathrm{DPPH}\right)-A\;\left(\mathrm{EtOH}\right)}\right)\times 100$$

where *A*_sample_ = absorbance in the presence of the compound and DPPH^•^, *A*_background_ = absorbance in the presence of the compound alone, *A*_DPPH_ = absorbance in the presence of DPPH^•^ radical solution, *A*_EtOH_ = absorbance of 96% ethanol.

##### 2,2′‐Azinobis‐(3‐Ethylbenzothiazoline‐6‐Sulfonic Acid) (ABTS^•+^) Assay

The ABTS^•+^ [2,2′‐azinobis‐(3‐ethylbenzothiazoline‐6‐sulfonic acid)] radical scavenging activity of test compounds was determined spectrophotometrically using the ABTS^•+^ cation decolorization assay, with absorbance measured at 734 nm.^[^ ^32^ ^]^ Trolox was used as a reference antioxidant compound. Briefly, an aqueous ABTS stock solution (6.7 mM) was mixed with potassium persulfate (2.44 mM) in equal volumes and incubated in the dark for 18 h to generate the ABTS^•+^ radical. The ABTS^•+^ working solution was prepared by diluting 1 mL of the stock radical solution in 60 mL of deionized water to obtain an absorbance of ≈0.7. Activator stock solutions (10 mM in DMSO) were serially diluted in 96% ethanol to final concentrations of 0.8–100 µM. For the assay, 100 µL of ABTS^•+^ solution was mixed with 100 µL of the test compound, and the decrease in absorbance was recorded after 5 min of incubation in the dark.

Antioxidant activity was expressed as % ABTS scavenging, calculated as follows

(3) $$\text{\%}\;\text{scavenging}=\left(1-\frac{A\left(\text{sample}\right)-A\;\left(\text{background}\right)}{A\left(\mathrm{ABTS}\right)-A\;\left(\mathrm{EtOH}\right)}\right)\times 100$$

where *A*_sample_ = absorbance in the presence of the compound and ABTS^•+^, *A*_background_ = absorbance in the presence of the compound alone, *A*_ABTS_ = absorbance in the presence of ABTS^•+^ radical solution, *A*_EtOH_ = absorbance of 96% ethanol with deionized water (1:1).

##### Lipid Peroxidation Assay

A fluorescent ratio probe, C11‐BODIPY^581/591^, was employed to assess the lipid peroxidation inhibition by compounds, as described by Drummen et al.,^[^ ^33^ ^]^ but with some modifications to an SR membrane system. Briefly, sarcoplasmic reticulum vesicles (0.3 mg mL^–^^1^) were labeled with C11‐BODIPY^581/591^ (5 µM) in HEPES buffer (pH 7.4) for 30 min at room temperature in the dark, followed by one washout at 37,000 g to remove excess free dye. Then, 100 µL of labeled SR membranes were added to 90 µL of test compounds (final concentration 10 and 30 µM) in a 96‐well Nunc black microplate (Thermo‐Fisher Scientific). Lipid peroxidation was initiated with 10 mM 2,2′‐azobis(2‐amidinopropane dihydrochloride) (AAPH) at 37 °C. Fluorescence of C11‐BODIPY^581/591^ was measured by simultaneous acquisition of the green (488/520 nm) and red signal (581/591 nm) after 30 min using a microplate reader (TECAN Infinite M 200, Switzerland). The ratio of green (oxidized) to red (reduced) fluorescence was used as an index of lipid peroxidation. Inhibition of lipid peroxidation % was calculated as follows

(4) $$\text{\%}\;\text{lipid peroxidation inhibition}=\left(1-\frac{F\left(\text{sample}\right)-F\;\left(\mathrm{SR}\text{unoxidized}\right)}{F\left(\mathrm{AAPH}\right)-F\left(\mathrm{SR}\text{unoxidized}\right)}\right)\times 100$$

where *F*_sample_ = fluorescence ratio (oxidized/reduced) in the presence of the compound and AAPH (test sample), *F*_AAPH_ = fluorescence ratio (oxidized/reduced) in the oxidized control (AAPH), *F*_SR unoxidized_ = fluorescence ratio in the negative control (SR membranes).

##### Statistical Analysis

Statistical comparisons were performed using an unpaired two‐tailed *t*‐test to assess the significance of SERCA1a ATPase activity protection from MGX‐induced inhibition by small‐molecule activators.

## Abbreviations

**Table:** 

ABTS	2,2′‐azino‐bis(3‐ethylbenzothiazoline‐6‐sulfonic acid)
AGEs	advanced glycation end‐products
BODIPY	boron‐dipyrromethene
C11‐BODIPY^581/591^	fluorescent lipid peroxidation probe
cLogD_7.4_	calculated distribution coefficient at pH 7.4
DPPH	2,2‐diphenyl‐1‐picrylhydrazyl
ER	endoplasmic reticulum
MGX	methylglyoxal
ROS	reactive oxygen species
SERCA	sarco/endoplasmic reticulum Ca^2+^‐ATPase
SR	sarcoplasmic reticulum
T2DM	type 2 diabetes mellitus
V_max_	Maximal velocity

## Conflict of Interest

The authors declare the following competing interest(s): Carlos Cruz‐Cortés and L. Michel Espinoza‐Fonseca are inventors on a provisional patent application that covers the fifteen synthetic SERCA activators reported in this paper (U.S. Provisional Patent Application 63/819,175).

## Author Contributions

**Carlos Cruz‐Cortés**: formal analysis: (equal); investigation (equal). **Silvia Micháliková**: formal analysis (supporting); investigation (equal). **Petronela Rezbáriková** : investigation (equal); funding acquisition (supporting). **L. Michel Espinoza‐Fonseca**: conceptualization (equal); formal analysis (equal); funding acquisition (equal); supervision (equal); writing—original draft (equal); writing review and editing (equal). **Jana Viskupičová** : conceptualization (equal); formal analysis (equal); funding acquisition (equal); supervision (equal); writing—original draft (equal); writing—review and editing (equal). **Carlos Cruz‐** **Cortés** and **Silvia** **Micháliková** contributed equally to this work.

## Supporting information

Supplementary Material

## Data Availability

The data that support the findings of this study are available from the corresponding author upon reasonable request.
