# Supplementary material for: Small‐Molecule Sarco/Endoplasmic Reticulum Ca2+‐ATPase Activators Reverse Methylglyoxal‐Induced Inhibition through Nonantioxidant Mechanisms
Source: ChemMedChem. 2025 Nov 30;21(1):e202500968. doi: 10.1002/cmdc.202500968 (PMC12716067; doi:10.1002/cmdc.202500968)
Supplement: Supplementary file 1 — Supplementary Material [file CMDC-21-e202500968-s001.pdf]

Supporting Information

Small-Molecule SERCA Activators Reverse Methylglyoxal-Induced Inhibition Through Non-Antioxidant Mechanisms

Carlos Cruz-Cortés,[1,‡] Silvia Micháliková,[2,‡] Petronela Rezbáriková,[2] L. Michel Espinoza-Fonseca,[1,*] and Jana Viskupičová[2,*]

[1] Center for Arrhythmia Research, Department of Internal Medicine, Division of Cardiovascular Medicine, University of Michigan, Ann Arbor, MI 48109, USA

[2] Centre of Experimental Medicine, Institute of Experimental Pharmacology & Toxicology, Slovak Academy of Sciences, 84104 Bratislava, Slovakia

‡These authors contributed equally to the work

*To whom correspondence should be addressed: jana.viskupicova@savba.sk (J.V.) or lmef@umich.edu (L.M.E.-F.)

This PDF file includes:

Supplementary Figures S1 and S2

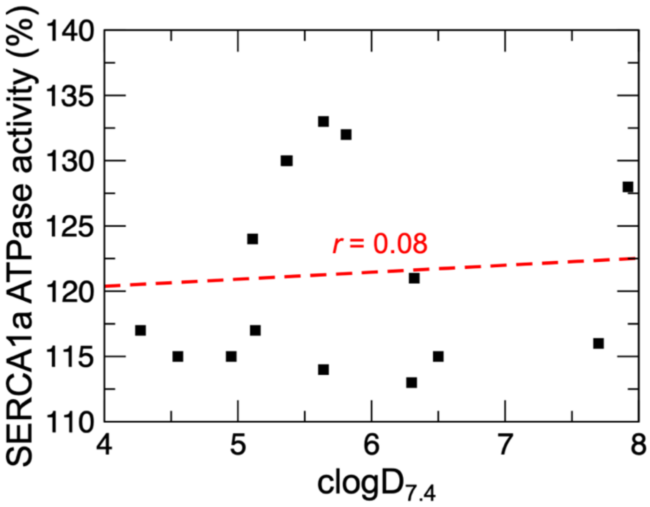

Figure S1. Correlation between SERCA1a activation and clogD$_{7.4}$. Scatter plot of SERCA1a ATPase activation (expressed as % activity relative to untreated control) versus calculated clogD$_{7.4}$ values. Each data point represents an individual compound. A linear regression model (red dashed line) was applied to evaluate the correlation between activation of ATPase activity and clogD$_{7.4}$ (r = 0.08).

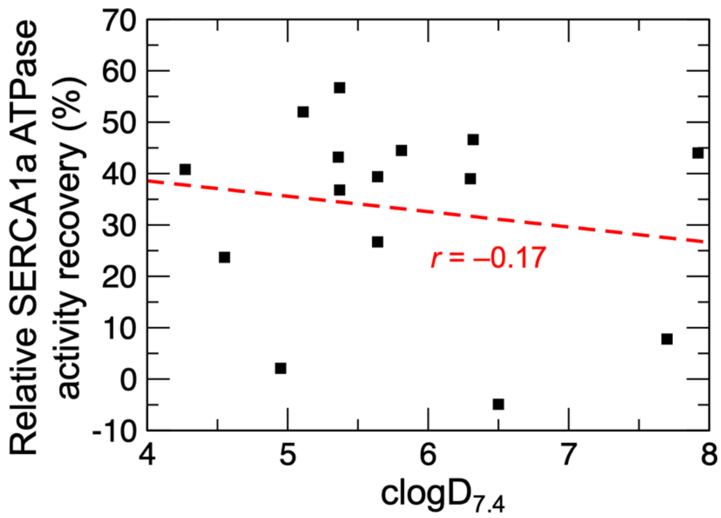

Figure S2. Correlation between recovery of SERCA1a activity and clogD$_{7.4}$. Scatter plot of recovery of SERCA1a activity (expressed as % relative recovery) versus calculated clogD$_{7.4}$ values. Each data point represents an individual compound. A linear regression model (red dashed line) was applied to evaluate the correlation between activation of ATPase activity and clogD$_{7.4}$ (r = –0.17).
